# Comparison of McGrath, Pentax, and Macintosh laryngoscope in normal and cervical immobilized manikin by novices: a randomized crossover trial

**DOI:** 10.1186/s40001-020-00435-0

**Published:** 2020-08-20

**Authors:** In Kyong Yi, Hyun Jeong Kwak, Kyung Cheon Lee, Ji Hyea Lee, Sang Kee Min, Jong Yeop Kim

**Affiliations:** 1 Department of Anesthesiology and Pain Medicine, Ajou University School of Medicine, 164, World cup-ro, Yeongtong-gu, Suwon, 16499 Korea; 2 Department of Anesthesiology and Pain Medicine, Gachon University, Gil Medical Center, 24, Namdong-Daero 774beon-gil, Namdong-gu, Incheon, 21565 Korea

**Keywords:** Laryngoscopy, Intubation, Videolaryngoscope, Cervical immobilization, Novice

## Abstract

**Background:**

The aim of this study was to compare tracheal intubation performance regarding the time to intubation, glottic view, difficulty, and dental click, by novices using McGrath videolaryngoscope (VL), Pentax Airway Scope (AWS) and Macintosh laryngoscope in normal and cervical immobilized manikin models.

**Methods:**

Thirty-five anesthesia nurses without previous intubation experience were recruited. Participants performed endotracheal intubation in a manikin model at two simulated neck positions (normal and fixed neck via cervical immobilization), using three different devices three times each. Performance parameters included intubation time, success rate of intubation, Cormack Lehane laryngoscope grading, dental click, and subjective difficulty score.

**Results:**

Intubation time and success rate during first attempt were not significantly different between the 3 groups in normal airway manikin. In the cervical immobilized manikin, the intubation time was shorter (*p* = 0.012), and the success rate with the first attempt was significantly higher (*p* < 0.001) when using McGrath VL and Pentax AWS compared with Macintosh laryngoscope. Both VLs showed less difficulty score (*p* < 0.001) and more Cormack Lehane grade I (*p* < 0.001) in both scenarios. The incidence of dental clicks was higher with Macintosh laryngoscope compared with McGrath VL in cervical immobilized airway (*p* < 0.001).

**Conclusions:**

McGrath VL and Pentax AWS did not show clinically significant decrease in intubation time, however, they achieved higher first attempt success rate, easier intubation and better glottis view compared with Macintosh laryngoscope by novices in a cervical immobilized manikin model. McGrath VL may reduce the risk of dental injury compared with Macintosh laryngoscope in cervical immobilized scenario.

*Trial registration*: ClinicalTrials.gov (NCT03161730), May 22, 2017

https://clinicaltrials.gov/ct2/hom

## Background

Tracheal intubation is a critical procedure for securing the patient’s airway in various situations. Success of tracheal intubation during the first attempt is important, because failure leads to serious complications including, permanent brain damage and death [1], and more than two attempts of intubation following failure of the first attempt may increase the morbidity [2]. Trained paramedics frequently perform tracheal intubation in a prehospital setting, with reported success rate less than that of experienced physicians [3]. In addition, the incidence of difficult tracheal intubation is significantly higher approximately 12% occurring outside the operating theater [4, 5] compared with 0.005% during elective operation settings [6]. Furthermore, significantly increased morbidity and mortality were observed outside the operating theater [7, 8].

Especially, in trauma patients, cervical immobilization is often routinely performed to minimize spinal cord injury, it adds to the failure rate of tracheal intubation using a Macintosh direct laryngoscope, as it hinders the visualization of the larynx by disrupting the alignment of the oral, pharyngeal and tracheal axes. Studies show that up to 50% of failed prehospital tracheal intubations were related to cervical immobilization [9–12]. Thus, novel techniques and devices for non-physicians performing tracheal intubations in a prehospital setting are needed to increase the success rate of the procedure.

Indirect videolaryngoscopes (VLs), in turn, offer specific advantages compared with Macintosh direct laryngoscopes during cervical immobilization, because they do not require the alignment of the oral–pharyngeal–tracheal axes to visualize vocal cords, as they utilizes a fiberoptic camera placed at the end of the device for direct visualization of the glottis [13]. Compared with the Macintosh laryngoscope, Pentax Airway Scope (AWS) and McGrath VL improve the laryngeal view and success rate of intubation, and reduce intubation difficulty in patients with simulated difficult airway [14, 15]. In the previous manikin study by novice airway managers, Pentax AWS and McGrath VL provided benefits for intubation compared with the Macintosh laryngoscope in normal and difficult airway scenarios [16, 17]. However, the result of time to intubation between these two VLs and Macintosh laryngoscope was not consistent in both human and manikin studies [18–20].

To date, there are few studies examining the relative efficacies of the McGrath VL, Pentax AWS and Macintosh laryngoscope with novice persons. The aim of this study was to compare tracheal intubation performance regarding the time to intubation, glottic view, difficulty and dental click, by novice personnel using McGrath VL, Pentax AWS and Macintosh laryngoscope in a normal and cervical immobilized manikin model.

## Methods

All study protocols were approved by our hospital’s Institutional Review Board and the current trial was registered in ClinicalTrials.gov (NCT03161730). Written informed consent was obtained from all participants. Thirty-five anesthesia nurses without previous intubation experience were included as participants. All participants watched a video regarding the operation of Macintosh laryngoscope, McGrath VL, and Pentax AWS before performing the procedure. Participants also received a lecture and demonstration of the procedures in a manikin model regarding endotracheal intubation, appropriate usage of each device, and Cormack Lehane laryngoscope grading system by an anesthesiologist who did not participate in this study.

Each participant performed endotracheal intubation in a manikin model (Laerdal Airway Management Trainer, Laerdal Medical, Stavanger, Norway) using three different devices (McGrath VL, Pentax AWS and Macintosh direct laryngoscope) in two different neck positions (normal neck sniffing position and fixed neck position by cervical immobilization), three times each. In all, all participants performed a total of 18 endotracheal intubations. The sequence of the six different scenarios (three devices in two manikin neck settings) was randomized for each participant using random number table (http://www.random.org). Cervical immobilization of the manikin was done using a rigid neck collar (Philadelphia, West Deptford, NJ, USA). A No. 3 blade was used for both Macintosh direct laryngoscope and McGrath VL and a malleable intubating stylet (Intubating Stylet, Covidien, Dublin, Ireland) was used with these devices. For the endotracheal tube, a tube with a cuff measuring 7.0 mm in internal diameter (Portex Ltd., Hythe, Kent, UK) was used. All procedures were performed in an empty operating theater.

The primary performance parameter was intubation time, measured by a separate observer with a timer. Intubation time was defined as the time from passage of the laryngoscope blade past the manikin teeth to successful ventilation using an Ambu bag. During a single trial, a maximum of three attempts could be made within 120 s. Attempt failure was defined as failure of lung inflation on Ambu bagging after tube insertion. Trial failure was defined as failure of intubation more than 3 attempts or intubation time more than 120 s regardless of number of attempts. Performance parameters included tooth injury, Cormack Lehane laryngoscope grading, and subjective difficulty score. An event was recorded as “tooth injury” when a click sound from the manikin teeth was heard during intubation. Each participant graded the laryngoscopic view during intubation using the Cormack Lehane grading system. The subjective difficulty of the whole procedure was graded using a numeric rating scale (NRS, 0; extremely easy to 10; extremely difficult) after the procedure.

## Sample size calculation and statistical analysis

The current study was designed as a cross-over study. Sample size was calculated using a power analysis. Standard deviation of intubation time values from previous studies was 18 s, and based on this value, significance was set for values more than 15 s [18]. Significance of 5% with 80% of power resulted in a total of 4 subjects (*α* = 0.05, *β* = 0.2). This study was a 3 × 2 cross-over design, thus requiring a minimum of 24 subjects. A total of 35 subjects were enrolled considering dropout and data loss. Statistical analysis was performed using SPSS 23.0 statistical package (IBM, Chicago, IL, USA). The Kolmogorov–Smirnov test was used to assess the normality of continuous variable distributions. Categorical data were analyzed using the χ^2^-test. And *p*-values < 0.05 were considered significant for three-group comparison, and *p*-values < 0.017 (= 0.05/3) were considered significant for the Bonferroni correction of two-group comparisons. Kruskal–Wallis test with Bonferroni’s correction was used to analyze non-normally distributed continuous variables. *p*-values of < 0.05 as determined using the Kruskal–Wallis test were considered significant.

## Results

### Participants demographics

**Fig. 1:**
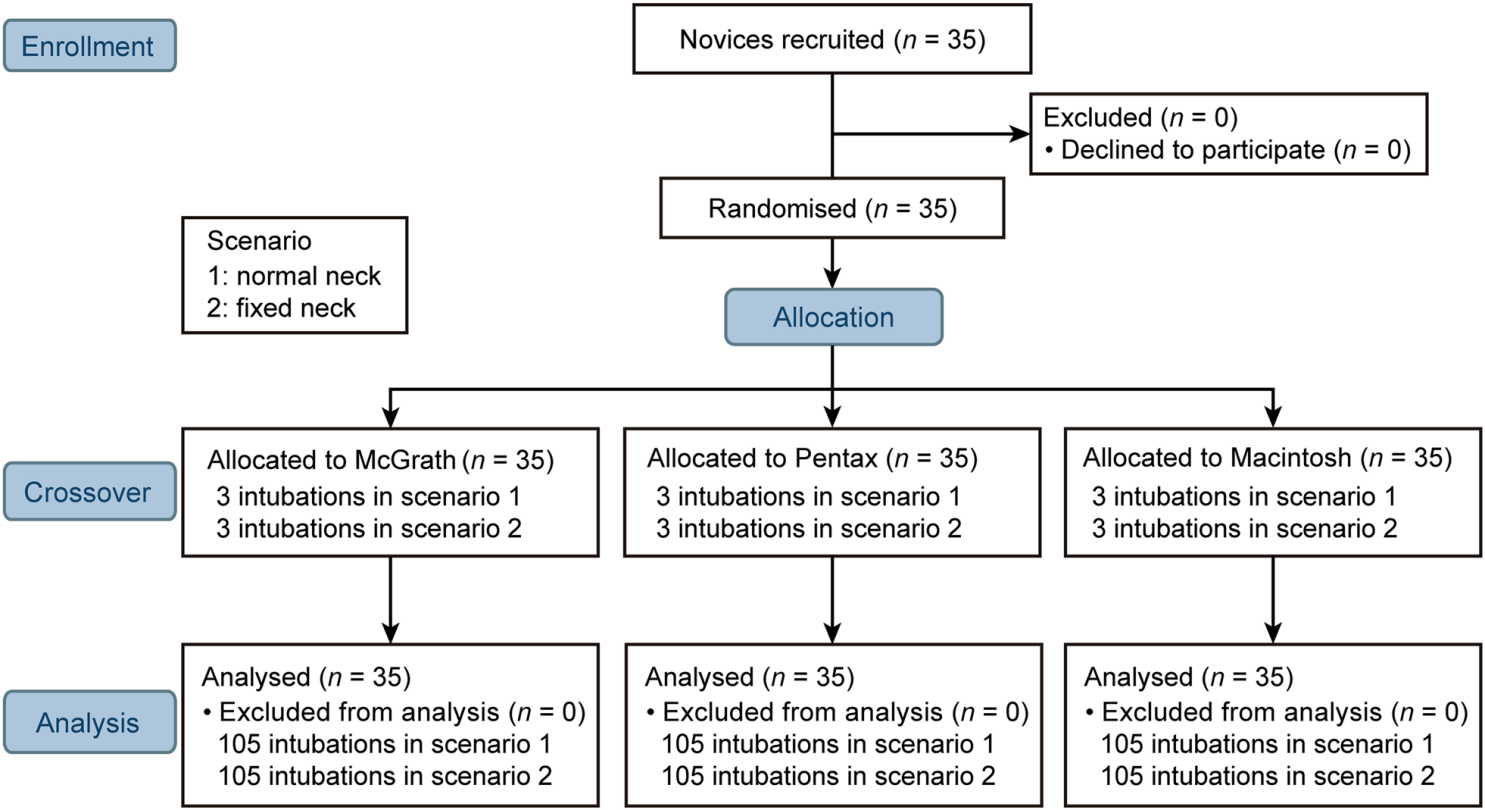
Consort diagram

A total of 35 anesthesia nurses completed the study. The participants included 8 males and 28 females and the mean ± standard deviation of their age was 29.2 ± 4.4 years (Fig. 1).

### Normal neck with sniffing position

Intubation time (median [interquartile range, IQR]) was similar with all three devices (19 [15–26] s, 19 [14–25] s and 19 [16–27] s in McGrath, Pentax and Macintosh groups, respectively, *p* = 0.497). Overall success rates (100%, 99% and 99% in McGrath, Pentax and Macintosh groups, respectively, *p* = 0.376) and success rates at the first attempt (99%, 100% and 96% in McGrath, Pentax and Macintosh groups, respectively, *p* = 0.376) were not significantly different between the groups. The incidence of dental clicks varied significantly between the groups (19%, 30% and 36% in McGrath, Pentax and Macintosh groups, respectively, *p* = 0.022). The Macintosh group showed a significantly higher incidence than McGrath group (*p* = 0.005). Difficulty grade (median [IQR]) varied significantly among the groups (3.0 [2.0–4.0], 3.0 [2.0–3.5] and 3.0 [2.0–5.0] in McGrath, Pentax and Macintosh groups, respectively, *p* = 0.013). Glottic grade varied significantly (*p* < 0.001) and Macintosh group had more grades of IIa, IIb and III compared to McGrath and Pentax groups (Table 1).

**Table 1:** Intubation parameters in the normal manikin

	McGrath (*n* = 105)	Pentax (*n* = 105)	Macintosh (*n* = 105)	*p* value
Total success rate	105 (100)	104 (99)	104 (99)	1.000
1st attempt success	104 (99)	105 (100)	101 (96)	0.376
Intubation time	19 [15–26]	19 [14–25]	19 [16–27]	0.497
Incidence of dental click	20 (19)	31 (30)	38 (36)^a^	0.022
Difficulty grade	3.0 [2.0–4.0]	3.0 [2.0–3.5]	3.0 [2.0–5.0]	0.013
Glottic grade				< 0.001
I	83 (79)	86 (82)	27 (26)	
IIa	7 (7)	1 (1)	17 (16)	
IIb	15 (14)	18 (17)	48 (46)	
III	0 (0)	0 (0)	10 (9)	
IV	0 (0)	0 (0)	3 (3)	

Values are number of patients (%) or median [interquartile range]. Intubation success, tracheal intubation performed within three attempts during 120 s; Intubation time, defined as the time from passage of the laryngoscope blade past the manikin teeth to successful ventilation using an Ambu bag; Dental click, when a click sound from the manikin teeth was heard during intubation; Difficulty grade, graded using a 11-point numeric rating scale (0; extremely easy–10; extremely difficult); Glottic grade, graded using the Cormack Lehane grading system

^a^*p* < 0.05/3, vs. McGrath group

### Fixed neck by cervical immobilization

Intubation time (median [IQR]) varied significant between the three devices (22 [15–34] s, 22 [13–33] s and 25 [18–40] s in McGrath, Pentax and Macintosh groups, respectively, *p* = 0.012). Overall success rates were not significantly different among the groups (100%, 100% and 98% in McGrath, Pentax and Macintosh groups, respectively, *p* = 0.331). However, success rates during the first attempt varied significantly between the groups (100%, 100% and 90.5% in McGrath, Pentax and Macintosh groups, respectively, *p* < 0.001) and first attempt failures included 10 cases in the Macintosh group. The incidence of dental clicks varied significantly between the groups (22%, 34% and 46% in McGrath, Pentax and Macintosh groups, respectively, *p* = 0.001) and Macintosh group had significantly higher incidence than McGrath group (*p* < 0.001). Difficulty grade (median [IQR]) varied significantly between the groups (3 [2–5], 3 [2–4] and 5 [3.5–7] in McGrath, Pentax and Macintosh groups, respectively, *p* < 0.001). Glottic grade was varied significantly (*p* < 0.001) and Macintosh group had more grades of IIb and III compared with McGrath and Pentax groups (Table 2).

**Table 2:** Intubation parameters in the cervical immobilized manikin

	McGrath (*n* = 105)	Pentax (*n* = 105)	Macintosh (*n* = 105)	*p*-value
Total success rate	105 (100)	105 (100)	103 (98)	0.331
1st attempt success	105 (100)	105 (100)	95 (91)	< 0.001
Intubation time	22 [15–34]	22 [13–33]	25 [18–40]	0.012
Incidence of dental click	23 (22)	36 (34)	48 (46)^a^	0.001
Difficulty grade	3 [2–5]	3 [2–4]	5 [3.5–7]	< 0.001
Glottic grade				< 0.001
I	77 (73)	83 (79)	13 (12)	
IIa	4 (4)	7 (7)	12 (11)	
IIb	24 (23)	15 (14)	64 (62)	
III	0 (0)	0 (0)	14 (13)	
IV	0 (0)	0 (0)	2 (2)	

Values are number of patients (%) or median [interquartile range]. Intubation success, tracheal intubation performed within three attempts during 120 s; Intubation time, defined as the time from passage of the laryngoscope blade past the manikin teeth to successful ventilation using an Ambu bag; Dental click, when a click sound from the manikin teeth was heard during intubation; Difficulty grade, graded using a 11-point numeric rating scale (0; extremely easy–10; extremely difficult); Glottic grade, graded using the Cormack Lehane grading system

^a^*p* < 0.05/3, vs. McGrath group

## Discussion

Our results showed that although McGrath VL and Pentax AWS did not show clinically significant decrease in intubation time, they achieved higher first attempt success rate and easier tracheal intubation compared with Macintosh direct laryngoscope by providing a better glottic view in cervical immobilized manikin model performed by novice intubators. In addition, McGrath VL reduced the dental click compared with Macintosh direct laryngoscope in cervical immobilized as well as in normal manikin models.

Indirect VLs have been reported to provide a better glottic view and thus lower difficulty scores of intubation [14]. In this study, both McGrath and Pentax groups provided a better glottic view and lower difficulty scores than Macintosh group in normal and in cervical immobilized positions. Because cervical immobilization by a rigid neck collar complicates the glottic view during intubation with a direct laryngoscope, these improvements by VLs were more pronounced under cervical immobilization in this study. Thus, both VLs in this study yielded faster tracheal intubation than Macintosh laryngoscope in a cervical immobilized manikin. By contrast, no differences in these intubation parameters were detected between three laryngoscopes in normal neck position. Our results are in accordance with previous studies [21, 22]. Choi et al. [21] have reported that McGrath VL provides higher first attempt success rate, faster intubation time and easier technique compared with Macintosh laryngoscope in cervical immobilized manikin performed by novice nurses. Pentax AWS also provided shorter intubation time and higher first attempt success rate compared to Macintosh laryngoscope in a similar scenario by novice paramedics [22]. Taken together, particularly, in a difficult airway situation, McGrath VL and Pentax AWS compared to Macintosh laryngoscope show advantages with novices in easiness and first attempt success rate in difficult airway situations. In our study, however, the average intubation time was approximately 3 s shorter for VLs, it failed to show differences over 15 s, which is considered to be clinically relevant time. Clinically significant differences in intubation time, therefore, cannot be proven from our results. Many previous studies showed that the result of time to intubation between these two VLs and Macintosh laryngoscope was not consistent in both human and manikin studies [18–20]. A recent meta-analysis of adult patients suggested that VLs reduce intubation failure and make intubation easier with experienced personnel, especially in patients with a predicted or known difficult airway. This study also showed that no evidence indicates whether VLs affect intubation time due to high level of statistical heterogeneity [23].

It is well known that a good laryngeal view with non-channeled VLs does not always guarantee successful tracheal intubation [24]. Successful intubation for non-channeled indirect VL requires effective eye–hand co-ordination and additional manipulations to steer the tube though the vocal cords using a malleable stylet while looking at the camera monitor. Meanwhile, Wetsch et al. [25] reported that channeled VLs provide faster intubation and decrease the failure rate compared with non-channeled VLs in a manikin simulating a trapped car accident by experienced anesthetist. However, Pieters et al. [26] reported that more intubation attempts are required by experienced and novice personnel when using channeled devices in manikins. In addition, in a recent analysis of King Vision VL utilizing both channeled and non-channeled blades, the non-channeled blade compared to channeled blade allowed faster intubation time with a similar first attempt success rate and glottic visualization in patients with normal airway by anesthetist [27]. The success of intubation depends on multiple factors, such as blade design (channeled or non-channeled); intubator skills (experienced or novice); patient airway (normal or difficult); and hospital setting (operating room or intensive care unit or prehospital) [28]. In our results, McGrath VL and Pentax AWS, compared to Macintosh laryngoscope show similar advantages with novices in terms of intubation time and first attempt success rate in difficult airway situations.

Regarding comparison of two indirect VLs, our results showed that a channeled VL (Pentax AWS) was subjectively easier to perform compared with a non-channeled VL (McGrath VL) in a cervical immobilized manikin model, without any differences in other parameters of intubation time and success rates at the first attempt. Studies comparing the performances of channeled and non-channeled VLs have shown inconsistent results. A previous study conducted in difficult simulated airways showed that Pentax AWS was quicker, easier and safer to intubate than GlideScope and Truview by a novice airway manager [16]. The bulky design of Pentax AWS blade may be associated with higher risk of dental injury than McGrath VL. In this study, the McGrath group showed only significant difference compared with the Macintosh group not with the Pentax group. Compared with manikin models, however, real cervical immobilized patients tend to result in limited mouth opening, with a mean inter-incisor distance less than 2 cm [11]. In such cases, use of thick, bulky devices result in difficult intraoral insertion and manipulation of the device, as well as higher chance of teeth injury. Channeled VL has generally a more bulky profile compared with non-channeled devices due to the tube guiding space. The thickness of the standard adult blade of a Pentax AWS is 18 mm, requiring an inter-incisor gap of ≥ 25 mm for smooth manipulation of the blade [29]. Studies incorporating newer thinner blades or even pediatric blades are needed in actual patients with difficult airway to investigate optimal design parameters of channeled devices.

The study limitations include the use of a manikin model, although extensively utilized in similar studies, which does not entirely reflect real-life clinical situations. The results of manikin studies may not be replicated in real patients due to several reasons. During intubation of real patients, various airway secretions may obstruct the view of the videolaryngoscope lens, rendering them useless [30]. Furthermore, although we have created a difficult airway situation via cervical immobilization in the manikin, there are numerous additional situations that comprise a difficult airway situation in real life, such as obesity, or a large tongue. Therefore, we should be cautious when applying the study results of manikin models to clinical situations. The setting in which the investigation was performed, a quiet empty operating room, is also quite different from a real clinical situation, where significant distraction and urgency are involved that may very well affect the performance of novice intubators. Similar studies performed under military combat settings or disaster settings, if feasible, are needed to investigate the true performance benefits of these devices by novice users.

## Conclusions

In conclusion, McGrath VL and Pentax AWS showed no clinical difference in intubation time, but higher first attempt success rate, easier intubation and better glottis view compared with Macintosh laryngoscope by novices in the cervical immobilized manikin model. McGrath VL may reduce the risk of dental injury compared with Macintosh laryngoscope.

## Data Availability

The datasets used and/or analyzed during the current study are available from the corresponding author on reasonable request.

## Abbreviations

AWS: Airway scope
IQR: Interquartile range
VL: Videolaryngoscope
